# A High-Regularity Porous SERS Substrate Prepared by Two-Step Mild and Hard Anodization for Sorbic Acid Detection

**DOI:** 10.3390/s26010156

**Published:** 2025-12-25

**Authors:** Chin-An Ku, Cheng-Hao Chiu, Chung-Yu Yu, Chuan-Yi Yang, Chen-Kuei Chung

**Affiliations:** Department of Mechanical Engineering, National Cheng Kung University, Tainan 701, Taiwan

**Keywords:** anodic aluminum oxide, AAO, high regularity, surface-enhanced Raman scattering, SERS, sorbic acid

## Abstract

Traditional colloid SERS substrates are mostly based on metal nanoparticles (MNPs), which have complex and time-consuming fabrication processes, poor structural control, and are susceptible to oxidation. As a result, solid-state SERS substrates have emerged as an effective alternative. Here, we propose using two-step mild and hard anodization to fabricate ordered anodic aluminum oxide (AAO) substrates with high total pore circumference for SERS detection. Hybrid pulse anodization (HPA) enables the fabrication of AAO at room temperature using 40 V in the first step and 40, 110, and 120 V in the second step of anodization. The different voltages applied in the second step effectively control the pore diameter, thereby achieving various nanostructures. The enhancement mechanism primarily originates from the high total pore circumference of nanostructures, which generates abundant hot spots around the pore peripherals, thereby significantly amplifying the SERS signal. Sorbic acid is a common preservative widely used in food products and employed as a test substance on high regularity AAO substrates at concentrations of 1000 ppm to 10 ppb. The resulting SERS spectra exhibited distinct characteristic peaks at 1640–1645 cm^−1^. The analytical enhancement factor is calculated as 1.02 × 10^5^ at the AAO substrate prepared by 110 V with the Si substrate as the reference. By appropriately tuning the process parameters, a limit of detection (LOD) as low as 10 ppb of sorbic acid was achieved.

## 1. Introduction

With the advancement of technology and increasing public awareness of food quality, food safety has become an important issue in recent years. Among food additives, five types of acid-based preservatives are commonly used, including benzoic acid, dehydroacetic acid, salicylic acid, propionic acid, and sorbic acid [1,2,3,4]. Currently, regulatory detection of preservatives mainly relies on high-performance liquid chromatography (HPLC), which is expensive, time-consuming, and unsuitable for large-scale screening. Although some studies have proposed improvements to HPLC [5,6], progress in reducing costs and time remains limited. Surface-enhanced Raman scattering (SERS) is a powerful detection technique [7,8,9,10,11], and all five types of acid-based preservatives can be inspected using SERS. Therefore, SERS holds great potential as an important alternative method for detecting food additives. However, traditional SERS substrates are mostly based on metallic nanoparticles (MNPs) [12,13,14,15,16,17,18], which involve complex and time-consuming fabrication processes, poor structural controllability, and susceptibility to oxidation. If the fabrication process cannot be further simplified, SERS could lose its advantage over HPLC in detection efficiency. Solid-state SERS substrates [19,20,21,22,23] can effectively address the issues of stability and reproducibility. However, the complexity and long fabrication time remain challenging. Furthermore, to compensate for the limited sensitivity of solid-state substrates, many research groups have developed three-dimensional nanostructures, such as nanocavities or V-shaped structures, to enhance performance [24,25,26,27,28]. Nevertheless, such approaches further extend the fabrication time of solid substrates to at least 12 h, and in some cases, also compromise their stability. Therefore, developing a stable, easily fabricated solid substrate with tunable nanostructures is the key to achieving reliable SERS detection of various substances.

Anodic aluminum oxide (AAO) is a well-known nanoporous template that can serve as an effective SERS substrate due to its stable and controllable surface morphology as well as electrochemical parameters [29,30,31]. Conventional AAO is typically fabricated using high-purity aluminum under low-temperature mild anodization through a two-step, time-consuming process [32,33,34,35,36], but its SERS signal performance is generally poor [37]. As a result, most studies have focused on depositing metallic nanoparticles onto AAO substrates for measurement. However, these limitations stem from the inefficient AAO fabrication process and the use of fixed voltages in both anodization steps, which lead to pore structures unsuitable for SERS substrates. In our previous work, we developed alloy-based SERS substrates with various nanostructures fabricated at room temperature (25 °C) and proposed the Pore Peripheral Plasmonic Mechanism (3PM) in studies of irregular-pore substrates [38]. Among multiple plasmonic modes [39,40,41], we have also demonstrated that metal sputtering onto a porous AAO substrate can generate strong localized surface plasmon resonance (LSPR) effects [38], thereby further enhancing the electromagnetic (EM) enhancement mechanism. We also demonstrated that AAO-based SERS substrates can effectively detect benzoic acid [23], which is a common preservative. Therefore, developing suitable substrates to establish a relationship between nanopore morphology and SERS signal intensity, and applying this to preservative detection, can greatly contribute to the advancement of solid-state SERS substrates and food safety research.

To address these challenges, we propose a two-step rapid MA–HA process to fabricate ordered nanoporous AAO solid SERS substrates with large total pore circumference (PC_total_) for sorbic acid detection. The method of achieving large pore diameters with small interpore distances through hybrid-pulse mixed mild-and-hard anodization has already been demonstrated using high-purity aluminum in our previous work [42]. In this study, we achieve similar results using a low-purity aluminum alloy and further apply this approach to enhance the SERS substrate signal. Compared with traditional AAO templates prepared at low temperatures under fixed voltages that produce small pores (<50 nm), our approach enables the formation of larger pores in the second high-voltage step, thereby increasing the number of hotspots. The interpore distance and pore diameter can be independently controlled by the first and second step voltages, respectively, resulting in AAO nanopores with high density, relative ordering, and large specific surface area. The 3PM mechanism indicates that the regions surrounding the pores are favorable for the generation of hotspots. Moreover, in our previous work on AAO-based humidity sensors, we also verified the importance of PC_total_ for sensing performance [43]. Larger PC_total_ indicates a higher proportion of available adsorption area relative to the substrate, which can effectively enhance sensor performance. This concept likewise presents opportunities for corresponding applications in SERS substrates. Therefore, creating nanostructures with more pore peripheries, meaning a larger PC_total_, can enhance the SERS signal and help establish the relationship between the nanostructure and SERS intensity.

## 2. Materials and Methods

**Figure 1:**
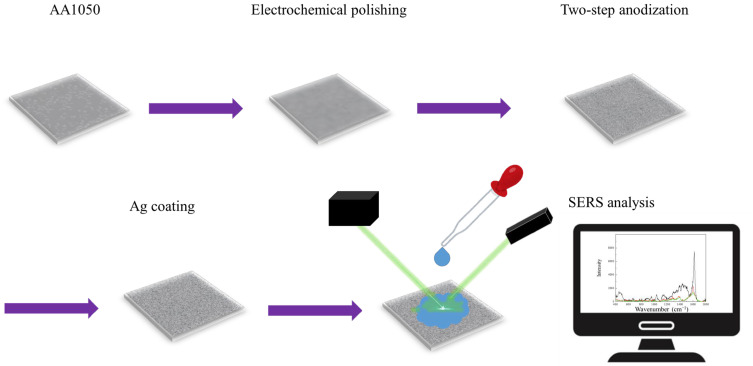
The process of AAO SERS substrate preparation using two-step mild and hard anodization.

The experimental process flow is shown in Figure 1. First, the commercial 1050 aluminum alloy (AA1050) (FAPO ENTERPRISE CO., LTD., Tainan, Taiwan) was cut into 2.5 cm × 2.5 cm. Then, AA1050 was electropolished by a two-step process to reduce surface scratches and roughness. The first step of coarse polishing was performed at 20 V in a mixture of HClO_4_:C_2_H_5_OH = 1:1 (v/v) for 1 min at 0 °C, and the second step of polishing was conducted in a HClO_4_:C_2_H_5_OH = 1:4 (v/v) solution under the same conditions for 5 min. Two-step anodization was performed in 0.3 M oxalic acid at 25 °C. In the first step of anodization, hybrid pulse anodization (HPA) was performed at 40/−2 V for 1 h. In the second step, HPA was applied at 40/−2 V for 1 h or at 110/−4 V, 120/−4 V for 10 min for comparison. The duty ratio of 40/−2 V was 5 s/5 s, and the ratio was 2 s/8 s for 110/−4 V and 120/−4 V. The formation of the AAO was achieved using a waveform generator with a voltage amplifier and a two-electrode electrochemical system, with platinum mesh as the counter electrode and the specimen as the working electrode. For surface morphology observation, a 12 nm Pt layer was coated on AAO. The nanostructure was observed using a high-resolution field scanning electron microscope (HRFESEM, HITACHI, SU-5000, Tokyo, Japan) and analyzed using the ImageJ software (ver. 1.53t). For SERS measurement, sorbic acid with concentrations from 1000 ppm to 10 ppb was used for detection, and 100,000 ppm on the Si substrate for analytical enhancement factor (AEF) calculation. The AAO substrates were fabricated into SERS substrates by sputtering approximately 20 nm of Ag using a coater. SERS measurements were performed using a 532 nm laser with an integration time of 1 s (GMDX, ProTrusTech, Tainan, Taiwan). The sorbic acid solution was dispensed onto the AAO SERS substrate using a pipette (LH0301002, FOUR E’s, Guangzhou, China), with a controlled droplet volume of 6 µL for measurement.

## 3. Results and Discussions

**Figure 2:**
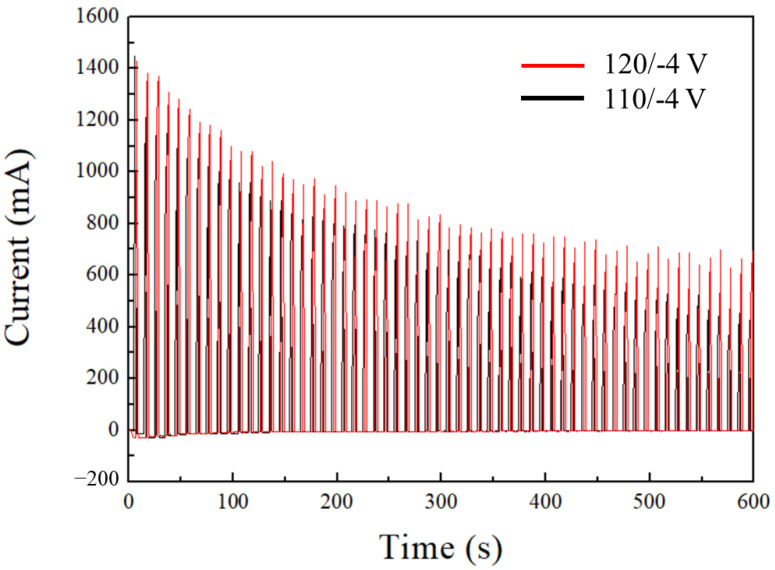
The current–time curves of the second step of the AAO anodization performed under high-voltage HPA conditions of 110/−4 V and 120/−4 V.

Figure 2 shows the current–time diagram of 110/−4 V and 120/−4 V during the second step of the anodization process. The black and red curves in the figure correspond to 110/−4 V and 120/−4 V, respectively, and anodization was carried out using the HPA method [29]. Initially, the current during the process was close to 1.4 A, followed by a gradual decline to stable ranges of 500 and 700 mA, respectively. The Joule heating accumulation was affected by the current value, and a higher current value resulted in a faster AAO growth rate with greater heat generation. Excessive heat accumulated during the reaction, leading to the destruction of AAO’s pore structure. Therefore, traditional HA processes are conducted at low temperatures to dissipate Joule heating and maintain the integrity of the nanopore structure. The HPA method with duty ratio modulation solves this problem and enables porous AAO to grow under high current density without burning. In the HPA method, a small negative potential is followed by a positive potential during anodization. These negative potentials counterbalance the reverse discharge caused by the capacitive characteristics of AAO, resulting in a negligible current during this phase. As a result, effective heat dissipation is achieved, allowing hard anodization to be performed at 25 °C. In the parameters used in this study, a duty ratio of 5 s:5 s was sufficient for effective heat dissipation at 40 V. However, in the HA range of 110 and 120 V, this duty ratio was inadequate to dissipate heat, leading to excessive Joule heating and the destruction of the nanopore structure. Therefore, in the second step of anodization, we adopted a duty ratio of 2 s:8 s to reduce heat accumulation and preserve pore integrity. However, partial burning due to excessive Joule heating can still be observed in the 120/−4 V sample.

**Figure 3:**
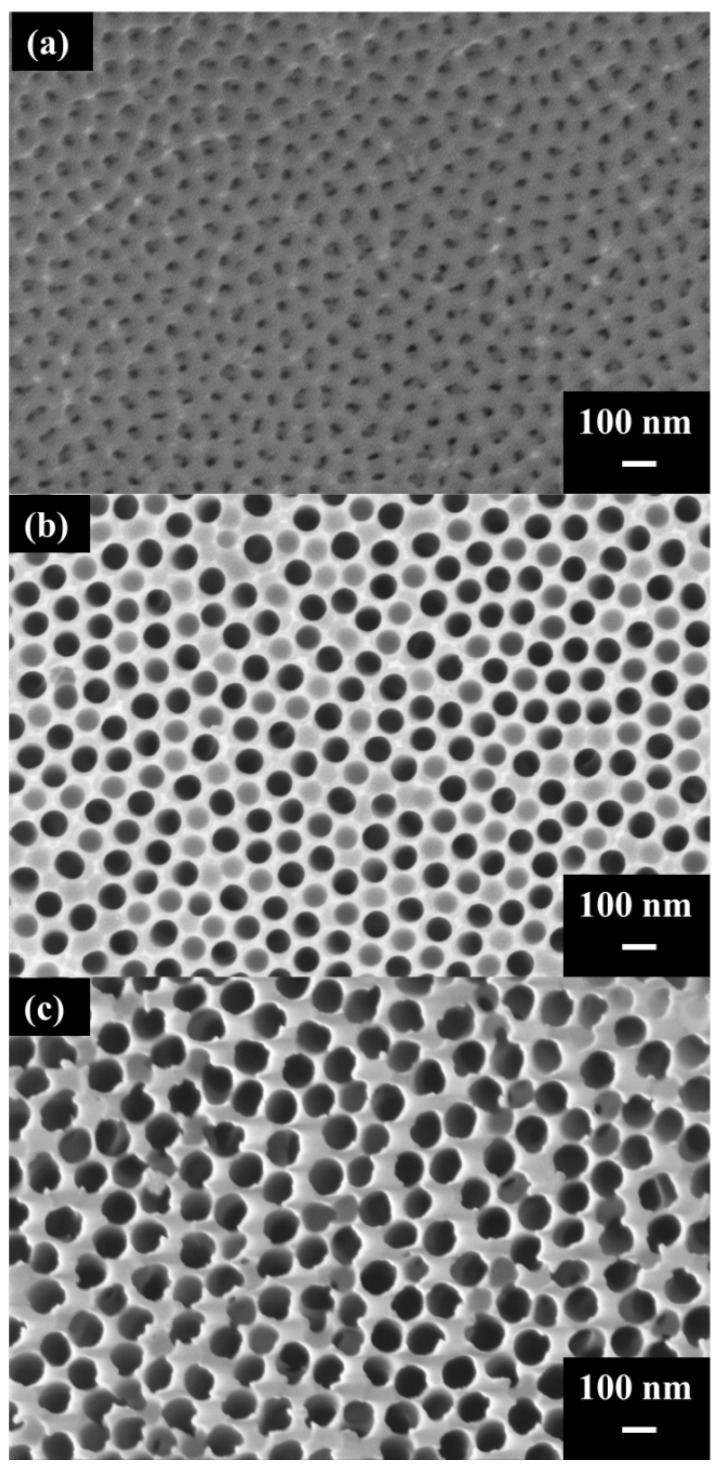
SEM images of AAO pores fabricated on 1050 aluminum alloy using the two-step MA–HA process. The first step was anodized at 40/−2 V for 1 h, followed by the second step conducted at (**a**) 40/−2 V for 1 h, (**b**) 110/−4 V for 10 min, and (**c**) 120/−4 V for 10 min.

Figure 3a–c present the surface SEM images of the second-step anodization conducted at 40/−2 V for 1 h and at 110/−4 V and 120/−4 V for 10 min. Through ImageJ analysis, the average pore diameters were calculated to be 37.4 ± 1.2 nm, 86.0 ± 0.8 nm, and 94.3 ± 3.5 nm, respectively. It can be observed that anodization at either 40/−2 V or 110/−4 V produces well-defined pores, with the latter exhibiting a noticeably smaller gap between pores, resulting in a higher PC_total_ structure. However, when the voltage is increased to 120/−4 V, excessive Joule heating causes pore structure burning, leading to partial pore collapse. Such a structure reduces the number of hotspot locations. Therefore, the AAO nanostructure formed at 110/−4 V provides the optimal hotspot density. In addition, the interpore distance of all three AAO structures is approximately 100 nm, which results from the 40 V anodization voltage used in the first step. The pre-pattern formed during the first step of anodization constrained the interpore distance, allowing the pore diameter to be adjusted solely through the second-step voltage [42]. This enabled the fabrication of high PC_total_ nanostructures while maintaining the same interpore distance.

**Figure 4:**
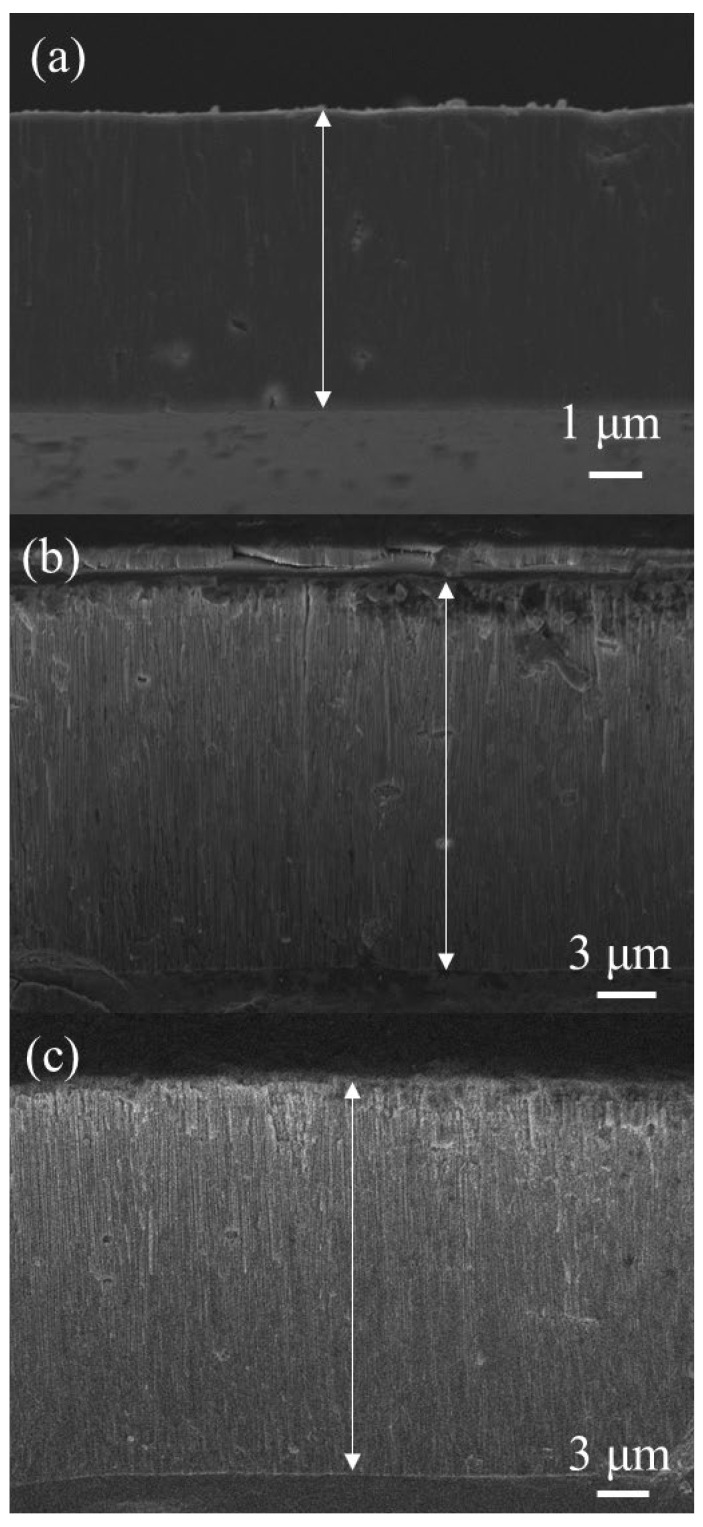
Cross-sectional SEM images of AAO fabricated at 25 °C using the two-step MA–HA process. (**a**) Anodization at 40/−2 V for 1 h, yielding a film thickness of 6 μm; (**b**) anodization at 110/−4 V for 10 min, yielding a film thickness of 18.5 μm; and (**c**) anodization at 120/−4 V for 10 min, yielding a film thickness of 21.5 μm.

Figure 4 shows the cross-sectional SEM images of AAO fabricated using the two-step MA–HA process, and Figure 4a–c correspond to anodization at (a) 40/−2 V for 1 h, (b) 110/−4 V for 10 min, and (c) 120/−4 V for 10 min. The film thicknesses are 6.0, 18.5, and 21.5 μm, respectively. It can be observed that the AAO growth rate under the high-voltage HPA process at 25 °C is remarkably fast, reaching 1.85 μm/min and 2.15 μm/min. Compared with the conventional AAO growth rate of less than 0.1 μm/min, this represents a more than 100 times improvement in fabrication efficiency.

**Figure 5:**
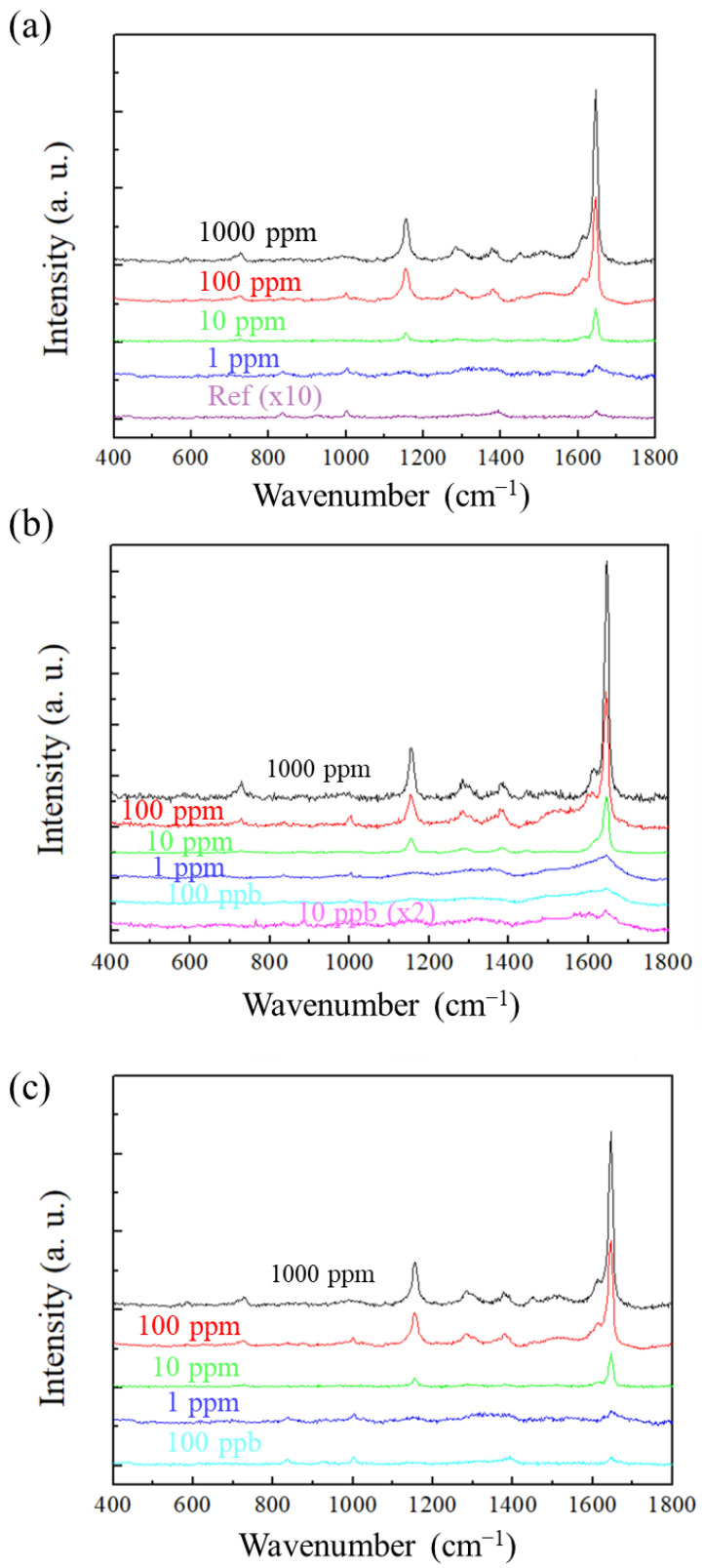
SERS detection results of sorbic acid at various concentrations using AAO SERS substrates fabricated under second-step anodization voltage: (**a**) 40/−2 V; (**b**) 110/−4 V; and (**c**) 120/−4 V.

Figure 5 shows the SERS spectra of sorbic acid measured using AAO substrates prepared at (a) 40/−2 V, (b) 110/−4 V, and (c) 120/−4 V. The black, red, green, dark blue, light blue, and pink curves represent concentrations of 1000 ppm, 100 ppm, 10 ppm, 1 ppm, 0.1 ppm, and 0.01 ppm, respectively. The highest peak of sorbic acid is at around 1640–1645 wavenumbers. The major peak position of sorbic acid has also been confirmed by other SERS studies that use sorbic acid as a test substance [4,44]. It is observed that the 110/−4 V substrate exhibits the strongest SERS signals, which is attributed to its larger pore structure, and facilitates the formation of more hotspots. Among the three parameters, the AAO substrate fabricated at 40/−2 V exhibits the poorest SERS sensing performance. This is because its pore peripheries and pore gaps provide fewer hotspots. Our previous studies have confirmed that the hotspots of AAO porous substrates originate from pore peripheries, gaps, and sharp tips. A larger number of pore peripheries is beneficial, while a smaller interpore distance is advantageous for hotspot generation. Since 2D pore structures with high circularity do not contribute sharp tips, the SERS intensity is determined solely by the pore peripheries and gaps. In Figure 5a, the SERS signal is weak, and the LOD for sorbic acid detection is 1 ppm. This indicates that, under the same interpore distance, porous structures with smaller pore diameters possess fewer peripheral and gap contributions, resulting in the lowest SERS signal. In contrast, the substrate fabricated by anodization at 110/−4 V shows the highest SERS signal (Figure 5b), because the larger pore diameter leads to the maximum enhancement of peripheral and gap hotspots, achieving an optimal LOD of 10 ppb for sorbic acid detection. For the substrate fabricated at 120/−4 V, the SERS signal decreases again (Figure 5c), because excessive Joule heating causes partial pore merging (Figure 3c). This burning-induced loss of pore gaps reduces the hotspots associated with the gaps, leading to a decline in signal. However, because the pore diameter is large, the contribution from pore peripheral remains substantial, so the SERS intensity is still higher than that of the substrate prepared by 40/−2 V. The AEF calculation is shown as Formula (1) below:

(1) $$AEF=\frac{C_{REF}}{C_{SERS}}\times \frac{I_{SERS}}{I_{REF}}$$

where C_SERS_ and C_REF_ are sorbic acid concentrations of 1000 ppm and 100,000 ppm on the two-step MA-HA AAO substrate prepared by 110/−4 V and the Si substrate; I_SERS_ and I_REF_ represent the peak intensities at 1640–1645 cm^−1^ of 1000 ppm sorbic acid on the AAO substrate and 100,000 ppm sorbic acid on Si, respectively. The AEF is calculated as 1.02 × 10^5^ with I_SERS_ = 46982 and I_REF_ = 46, respectively.

**Figure 6:**
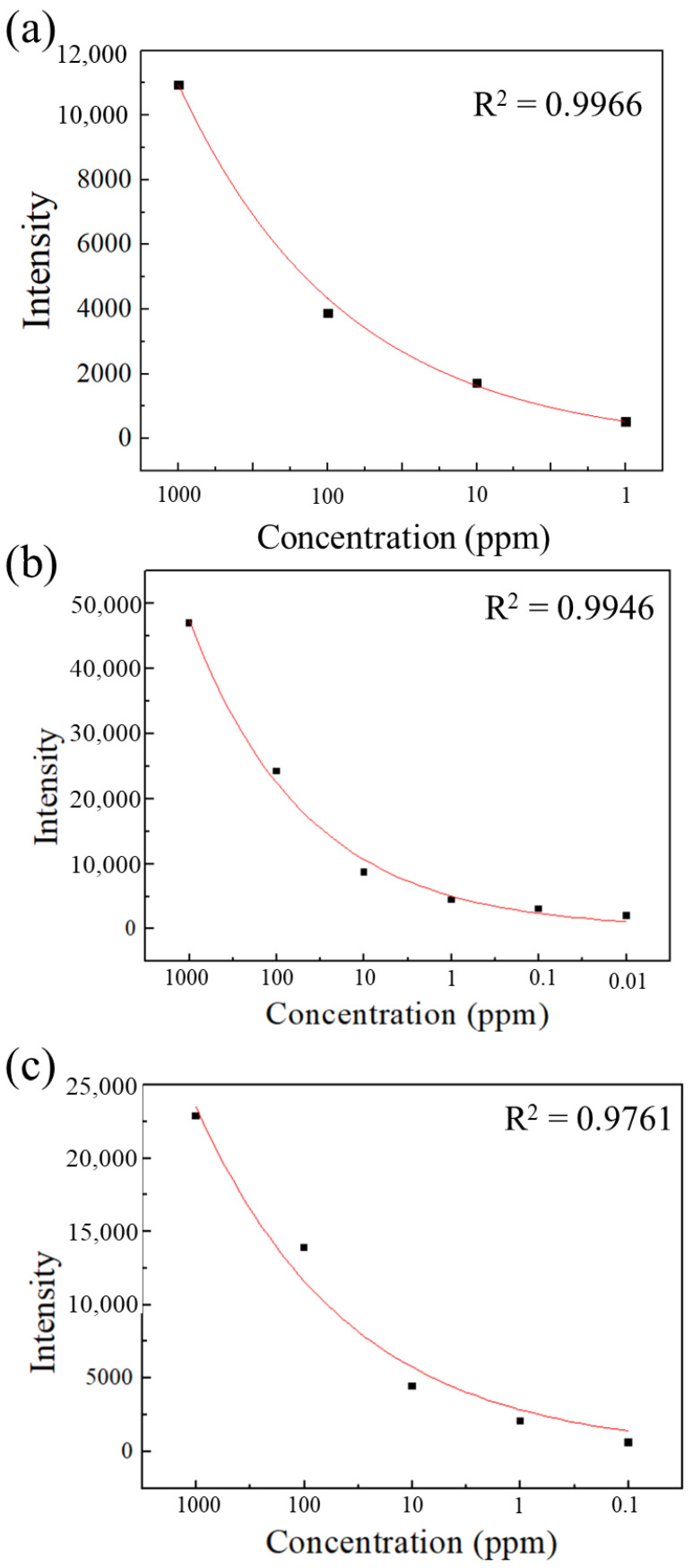
Exponential function fitting curves of sorbic acid on AAO substrates prepared at (**a**) 40/−2 V, (**b**) 110/−4 V, and (**c**) 120/−4 V.

Figure 6a–c present the fitting curves corresponding to the SERS spectra of sorbic acid. The plots are based on the peak intensity at approximately 1640–1645 cm^−1^. The fitted curves were generated using automatic exponential function analysis, yielding R^2^ values of 0.9966, 0.9946, and 0.9761 for AAO substrates fabricated at 40/−2 V, 110/−4 V, and 120/−4 V, respectively. These results indicate high consistency between the measured data and the fitted predictions, with a strong correlation between spectral intensity and concentration. The relatively lower R^2^ value of 0.9761 in Figure 6c may be attributed to the irregularities caused by pore damage in this sample.

**Figure 7:**
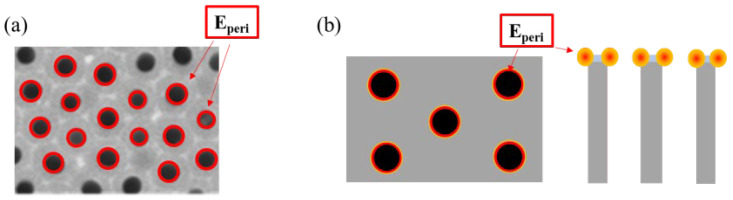
Schematic diagram of hotspots on the AAO SERS substrate: (**a**) the SEM image, and (**b**) front-view and cross-sectional schematics.

Figure 7 shows a schematic illustration of the hotspot locations on the AAO substrates prepared using the two-step MA–HA process. In Figure 7a, the hotspots around the pore periphery (E_peri_) are marked on the SEM image, while Figure 7b presents schematic hotspot locations observed from both the top and cross-sectional views. The SERS hotspots of AAO-based substrates are generally located around the pore periphery. Therefore, it is expected that the SERS signal intensity is positively correlated with the total pore circumference (PC_total_) within a given area, as demonstrated by the following equation:

(2) $${PC}_{total}=n\times D_{p}\times \pi$$

where n is the number of pores in a certain area, and D_p_ represents the diameter of the AAO pore. We previously demonstrated that the concept of total pore circumference can effectively predict the response of AAO-based inductive sensors. For AAO used as a SERS substrate, hotspots are primarily generated around the pore periphery. Therefore, the SERS performance is highly correlated with the total pore circumference. In our parameter design, the first step of anodization for AAO was conducted at 40/−2 V for all samples. The pre-texture formed at this voltage constrains the interpore distance of AAO, so the interpore distance remains at approximately 100 nm after the second step of anodization. It indicates that the number of pores within a given area is the same among the three substrates; in other words, n is identical for all three. Therefore, the PC_total_ of the AAO substrate within a given area is directly determined by the pore diameter. From the SEM images, it is evident that the total circumference for the 110/−4 V condition is significantly greater than that of the 40/−2 V condition, resulting in the strongest sensing signal. In contrast, the AAO substrate fabricated at 120/−4 V shows poor signal performance due to pore collapse and interconnection caused by Joule heating. Although AAO fabricated at 120/−4 V exhibits a larger pore diameter, the collapse and structure burning of pores still result in poorer signal enhancement. The electric field effects of the AAO substrates were also compared and simulated using COMSOL (ver. 6.3), as shown in Figure S1. The simulation results are consistent with the experimental data, showing that the AAO substrate fabricated at 110 V exhibits the highest electric field intensity. In addition, the hot spots are concentrated around the pore edges and interpore gaps, which is in agreement with the proposed mechanism. From our experimental data, it is verified that larger pore diameters are advantageous as long as the pores do not collapse. This further confirms that increasing the PC_total_ has a positive impact on AAO-based sensors. With a sufficient number of hotspot sites, the AAO substrate fabricated at 110/−4 V achieves the best LOD at 10 ppb for sorbic acid.

## 4. Conclusions

We successfully fabricated ordered AAO-based SERS substrates using AA1050 at 25 °C through a two-step hybrid pulse MA–HA anodization process. Unlike the conventional low-temperature two-step anodization conducted at constant voltage, our process not only improves fabrication efficiency but also enables access to more diverse AAO structures. We propose tuning the duty ratio of the HPA, which allows high-voltage anodization at 110–120 V. The resulting growth rate reaches 1.85–2.15 µm/min, which is over 100 times faster than the traditional rate (<0.1 µm/min). By applying different voltages during the two steps, the interpore distance and pore diameter can be independently controlled. Achieving a larger pore diameter under the same interpore distance effectively increases the total circumference and the number of hotspots, which is beneficial for SERS detection. We found that the SERS substrate anodized at 40/−2 V in the first step and 110/−4 V in the second step achieved an AEF of 1.02 × 10^5^ for 1000 ppm sorbic acid. In addition, its high-circumference structure enabled detection of sorbic acid down to 10 ppb. This efficient 2D porous AAO substrate can be further applied to the detection of preservatives, antibiotics, and toxic substances in the future.

## Data Availability

The data are presented in the coauthors’ research results and schematic drawings, available on request.

## Supplementary Materials

The following supporting information can be downloaded at https://www.mdpi.com/article/10.3390/s26010156/s1. Figure S1: COMSOL simulation of AAO substrate prepared at (a) 40 V, (b) 110 V, and (c) 120 V in 2^nd^ anodization.
