# Supplementary material for: A High-Regularity Porous SERS Substrate Prepared by Two-Step Mild and Hard Anodization for Sorbic Acid Detection
Source: Sensors (Basel). 2025 Dec 25;26(1):156. doi: 10.3390/s26010156 (PMC12787714; doi:10.3390/s26010156)
Supplement: Supplementary file 1 [file sensors-26-00156-s001.zip › sensors-4042095-supplementary.pdf]

Supplementary Files

A high-regularity porous SERS substrate prepared by two-step mild and hard anodization for sorbic acid detection

Chin-An Ku, Cheng-Hao Chiu, Chung-Yu Yu, Chuan-Yi Yang and Chen-Kuei Chung*

Department of Mechanical Engineering, National Cheng Kung University, Tainan 701, Taiwan;

* Correspondence: ckchung@mail.ncku.edu.tw

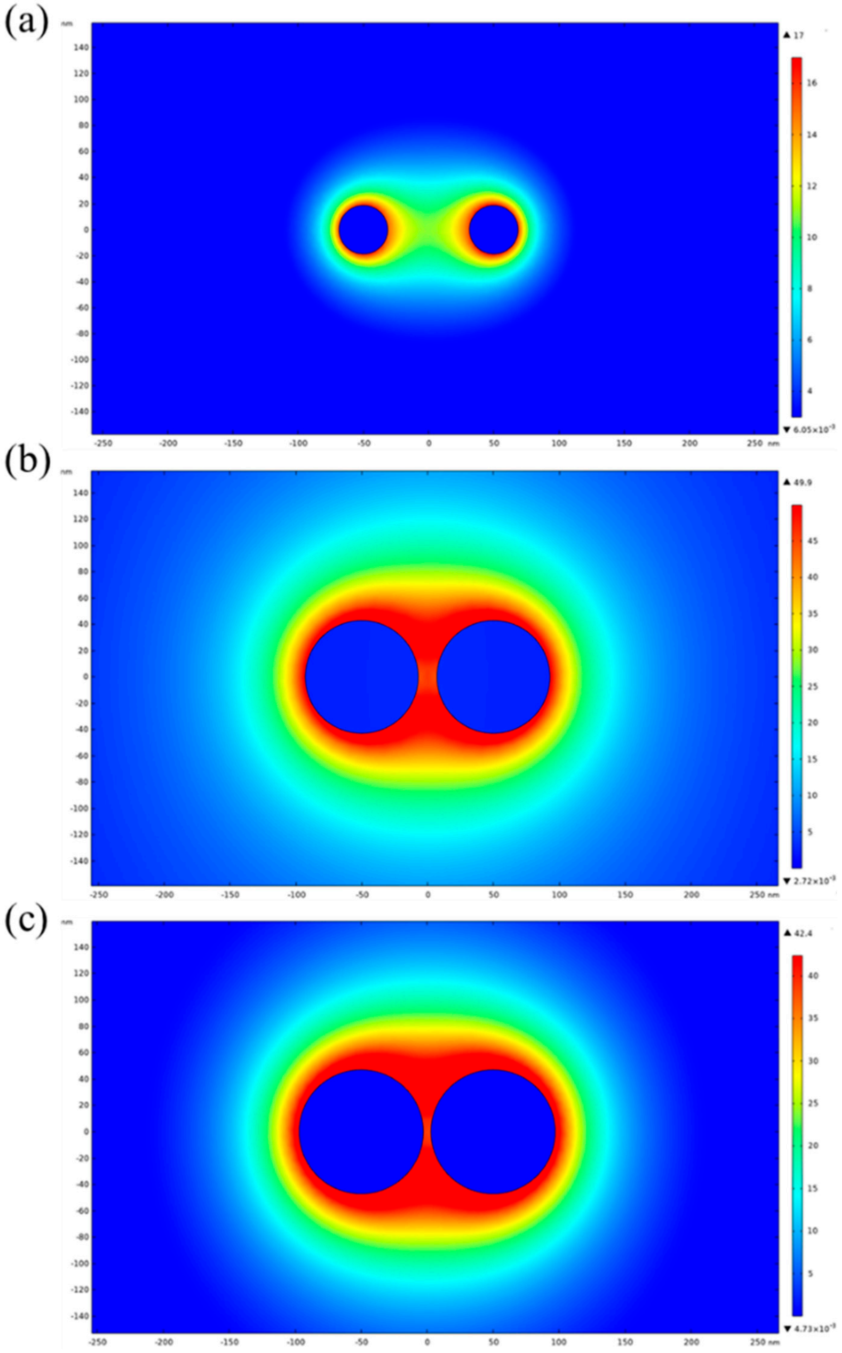

Figure S1 COMSOL simulation of AAO substrates prepared at (a) 40 V, (b) 110 V, and (c) 120 V during 2nd anodization.

Figure S1 presents the simulated electrical field of AAO fabricated at (a) 40 V, (b) 110 V, and (c) 120 V at 2nd step anodization. For AAO prepared at (a) 40 V, (b) 110 V and (c) 120 V, 2 pores with diameter of 37.4, 86.0 and 94.3 nm and interpore distance of 100 nm are presented. The COMSOL simulation is carried out by frequency-domain calculation with Drude-Lorentz model, and a plane wave is polarized to excite the surface plasmons. The parameters of material properties such as Ag, air, and Al_2O_3 follow the default in the software. The corresponding maximum electric field strengths are 17, 49.9, and 42.4 V/m, respectively, which are consistent with the hypotheses proposed in the manuscript. The simulation results further reveal that the electric field is indeed concentrated around the pore peripheral and gaps, in agreement with our proposed mechanism. Although the electric field intensities in Fig. S1 (b) and (c) are similar, the simulated pore morphologies cannot fully capture pore-wall burning effects observed in the SEM images. As a result, we believe that the electric field strength in Fig. S1 (c) may be slightly overestimated. To sum up, these results confirm that the proposed mechanism, experimental data, and simulation outcomes are in good agreement.
